# Editorial: Ischemic stroke management: From symptom onset to successful reperfusion and beyond

**DOI:** 10.3389/fneur.2022.1042342

**Published:** 2022-10-13

**Authors:** Peter B. Sporns, Johanna M. Ospel, Marios-Nikos Psychogios

**Affiliations:** ^1^Department of Neuroradiology, Clinic for Radiology & Nuclear Medicine, University Hospital Basel, Basel, Switzerland; ^2^Department of Diagnostic and Interventional Neuroradiology, University Medical Center Hamburg-Eppendorf, Hamburg, Germany

**Keywords:** stroke, thrombectomy, endovascular thrombectomy (EVT), imaging, reperfusion

## Introduction

Fast and complete reperfusion of the occluded vessel territory is the key to every revascularization therapy in stroke patients, no matter if treated with alteplase or endovascular thrombectomy (EVT) (1–4). However, there is room for substantial improvement in time efficiency and techniques to achieve reperfusion [(3, 5), Advani]. This introduction of the Research Topic “Ischemic Stroke Management: From Symptom Onset to Successful Reperfusion and Beyond” left room for a wide variety of topics for articles, which is reflected by a large number of high-quality articles published in this Research Topic (59). The predefined areas of interest included—but were not limited to—the one-stop management of ischemic stroke patients in the angio-suite, novel methods of pre-hospital patient triage, new procedural techniques and software solutions for effective patient triage, clinical consequences of improved time metrics and prediction of functional outcomes following hyperacute reperfusion therapies. The aim of the Research Topic was to investigate the impact of logistical and procedural improvements on the success of reperfusion and the clinical outcome of ischemic stroke patients.

Looking at the studies published in this Research Topic and starting with pre-hospital triage optimization Cabal et al. report that their new prehospital triage test (FAST PLUS) yielded significant reductions of onset-to-groin times in patients receiving EVT, meaning that median onset-to-groin times reduced from 213 to 142 min in their cohort from the Czech Republic. Weissenborn et al. analyzed workflow and outcome metrics of stroke patients undergoing EVT in their German tertiary stroke center as a starting point for optimization. In their analysis, they found several factors leading to a delay in treatment (i.e., medical treatment of a hypertensive crisis, epileptic fits, vomiting, or agitation, repeated brain imaging, and transfer from other hospitals). Hence, they concluded that analyses of workflow and treatment results should be carried out regularly to identify the potential for optimization of operational procedures and selection criteria for patients who could benefit from EVT (Weissenborn et al.).

At least as important as prehospital triage and procedural optimization are the technical results of the thrombectomy procedure itself (5, 6). Thus, various articles in this Research Topic investigated technical and procedural developments. In their retrospective study, Guenego et al. described the impact of clot shape on successful middle cerebral artery M1-segment endovascular reperfusion and found that clot shape as determined on T2^*^ imaging, appears to be a predictor of successful reperfusion after EVT because angulated and bifurcating clots were associated with poorer rates of successful reperfusion. Moreover, Candel et al. found that the size of stent retriever matters in acute M1 occlusions treated with aspiration-assisted mechanical thrombectomy. A longer stent retriever with a larger nominal diameter achieved a higher complete and successful first pass effect and higher successful reperfusion compared to a shorter stent retriever (Candel et al.). Another analysis by Etter et al. found that application of a new coating to the delivery wire of the Trevo retriever, with the new device being called the “Trevo NXT” stent retriever, was an effective and safe tool for EVT that could be more easily deployed and was especially effective when used for combined approaches. When looking at the definition for successful recanalization of the thrombectomy procedure, Yoo et al. reported that in their international multicenter trial, first-pass excellent reperfusion (defined as TICI 2c-3), was the technical revascularization endpoint that best predicted functional independence and concluded that this should be an angiographic endpoint for future trials, further consolidating prior evidence from published studies.

Previous studies have shown that histological thrombus composition impacts procedural and technical outcomes of EVT, that thrombus composition is associated with stroke etiology and that the thrombus composition itself can be predicted from admission imaging (7–11). In this issue, Eto et al. report that atherosclerotic components in retrieved thrombi might provide useful clues for diagnosing stroke pathogenesis. Their investigation of the association between onset-to-imaging time and radiological thrombus characteristics suggested that elapsed time from stroke onset plays a limited role in the interpretation of radiological thrombus characteristics and their effect on treatment results and should therefore not confound imaging-based thrombus analysis, at least in the early time window (Tolhuisen et al.). Regarding the visualization of thrombus content, LaGarange et al. reported that MicroCT can be used as an indicator for red blood cells-rich composition of clots, and a combination of MicroCT and electron microscopy revealed further valuable information with regard to clot composition.

Regarding the ongoing debate of intravenous thrombolysis plus EVT vs. EVT alone, Maier et al. report that in patients included in the German Stroke Registry, bridging IVT improved rates of successful reperfusion and long-term functional outcome in mothership patients with anterior circulation large vessel occlusion, which is in line with the results of the recently published SWIFT DIRECT trial. This was further confirmed by a meta-analysis concluding that bridging thrombolysis provides more benefits than EVT alone in terms of clinical functional outcomes without compromising safety in AIS patients with LVOs (Li et al.).

Furthermore, several studies in this article collection further investigated indication criteria in special populations, which were not represented by randomized trials. For example, Kastrup et al. reported that in dependent patients, EVT led to less patients with poor outcomes and smaller infarcts compared to intravenous thrombolysis alone.

## Discussion and future challenges

The collection of articles in this Research Topic contributes to the continuous evolvement of further defining patient subgroups that will benefit from hyperacute reperfusion therapies. As an example, there are three currently ongoing randomized controlled trials investigating the benefit of EVT in patients with medium vessel occlusions (DISTAL, NCT05029414, ESCAPE-MeVO, NCT05151172, and DISCOUNT, NCT05030142). Defining imaging and clinical characteristics to identify potential EVT candidates within this patient subgroup will help to treat as many stroke patients as possible with the game-changing endovascular thrombectomy but, on the other hand, also help to prevent harming patients, who are very unlikely to benefit. Further logistic and procedural improvements will pave the way toward treating patients even more effectively and in the end find the optimal and fastest therapy for individual stroke patients.

## Author contributions

All authors drafted and revised this editorial. All authors contributed to the article and approved the submitted version.

## Conflict of interest

The authors declare that the research was conducted in the absence of any commercial or financial relationships that could be construed as a potential conflict of interest.

## Publisher's note

All claims expressed in this article are solely those of the authors and do not necessarily represent those of their affiliated organizations, or those of the publisher, the editors and the reviewers. Any product that may be evaluated in this article, or claim that may be made by its manufacturer, is not guaranteed or endorsed by the publisher.
